# Self‐regeneration of an extensive bony defect following mandibular resection

**DOI:** 10.1002/ccr3.3057

**Published:** 2020-06-22

**Authors:** Mahboube Hasheminasab, Reza Sharifi, Mahsa Mortazavi, Arezoo Javani

**Affiliations:** ^1^ Department of Oral and Maxillofacial Surgery Shariati Hospital Tehran University of Medical Sciences Tehran Iran; ^2^ Craniomaxillofacial Research Center (CMFRC) Shariati Hospital Tehran University of Medical Sciences Tehran Iran; ^3^ Department of Orthodontics University of the Pacific Arthur A Dugoni School of Dentistry San Francisco California USA; ^4^ Department of Oral and Maxillofacial Surgery School of Dentistry Babol University of Medical Sciences Mazandaran Iran

**Keywords:** bone regeneration, mandibular defect, mandibular resection, self‐repair

## Abstract

Spontaneous bone regeneration is a rapid and uncommon formation of new bone in a previous bone defect. Preservation of periosteum as the main source of osteogenesis, young age, and genetics are possible important factors related to this phenomenon.

## Graphical abstract

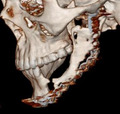

## 1 INTRODUCTION

Spontaneous bone regeneration has been reported following mandibular resection in both growing patients and adults. The phenomenon has been observed in small and wide mandibular defects, even in total mandibulectomies. In most of the reported cases, mandibular body or ramus showed rapid spontaneous regeneration.^1^ Bone has a unique potential to self‐repair and regenerates following trauma. Regenerated tissue is comparable to that of the original tissue without leaving a scar.^2^, ^3^ The mechanism of this healing pattern is often explained by the constant dynamics of the bone resorption and apposition in normal bone considering recapitulation of embryonic osteogenesis and growth.^3^, ^4^

Although this phenomenon is unexpected, the success of bone healing is related to the size of the defect, the anatomical location, the patient's age, and other parameters such as presence of an intact periosteum, infection, stabilization of the remaining mandibular segments,, and genetics.^4^, ^5^, ^6^

The present study reports spontaneous bone regeneration in a 21‐year‐old male after an almost hemi‐mandibulectomy due to odontogenic myxoma.

## 2 CASE HISTORY

**Figure 1:**
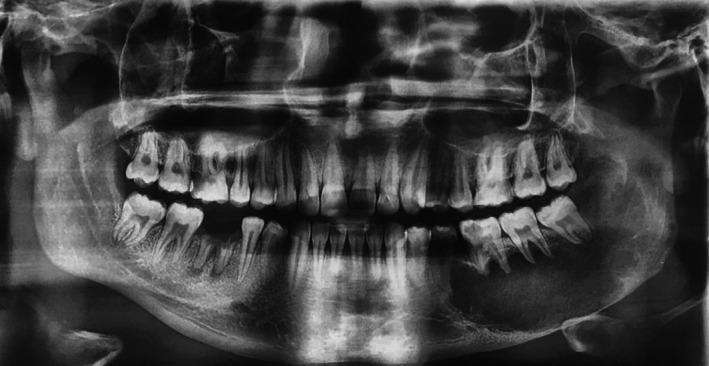
Preoperative panoramic view. An ill‐defined radiolucency extending from right second premolar to the right mandibular ramus is evident

A 21‐year‐old male was referred to the Department of Oral & Maxillofacial Surgery of Tehran University of Medical Sciences, Tehran, Iran, complaining of an asymptomatic swelling in the right mandible while an incisional biopsy was performed. The lesion was diagnosed with odontogenic myxoma. On clinical examination, a nontender, expansile mass of the right mandibular body, and ramous was observed. The overlying mucosa was normal, and no sign of infection was detected. The panoramic radiograph showed an ill‐defined radiolucency extending from right second premolar to the right mandibular ramus (Figure 1).

## 3 INVESTIGATIONS AND TREATMENT

**Figure 2:**
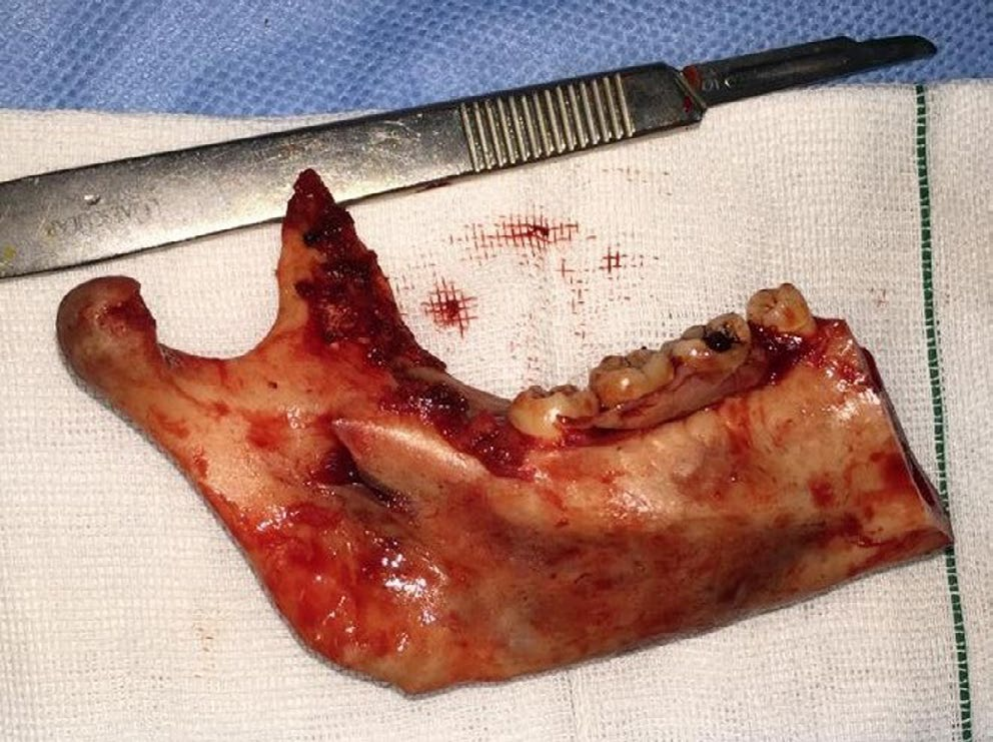
The resected segment of mandible

A cone beam computed tomography scan revealed an expansile mass leaving thin buccal and lingual cortices. The patient was scheduled for hemi‐mandibulectomy based on clinical findings and histopathological report. Mandibular body and ramus were approached via an extra oral submandibular incision. Dissection on mandible was carried out through the subperiosteal plan, with gentle manipulation of periosteum on buccal and lingual sides. Osteotomy line was designed distal to mandibular right canine considering 1 cm safe margin for the lesion. After complete muscular detachment, mandibular right condyle was disarticulated from the glenoid fossa (Figure 2). A condylar reconstruction plate was previously formed based on 3D stereolithographic model of the mandible. The sterilized pre‐pent condylar reconstruction plate was used to preserve facial contour. The wound was closed tension free in three layers, and a vacuum drain was inserted. Subsequent postoperative recovery was uneventful. Declaration of Helsinki on medical protocol was followed, and informed consent was obtained. The study protocol was reviewed and approved by the clinical research ethics board of Tehran University of Medical Sciences.

## 4 OUTCOME AND FOLLOW‐UP

**Figure 3:**
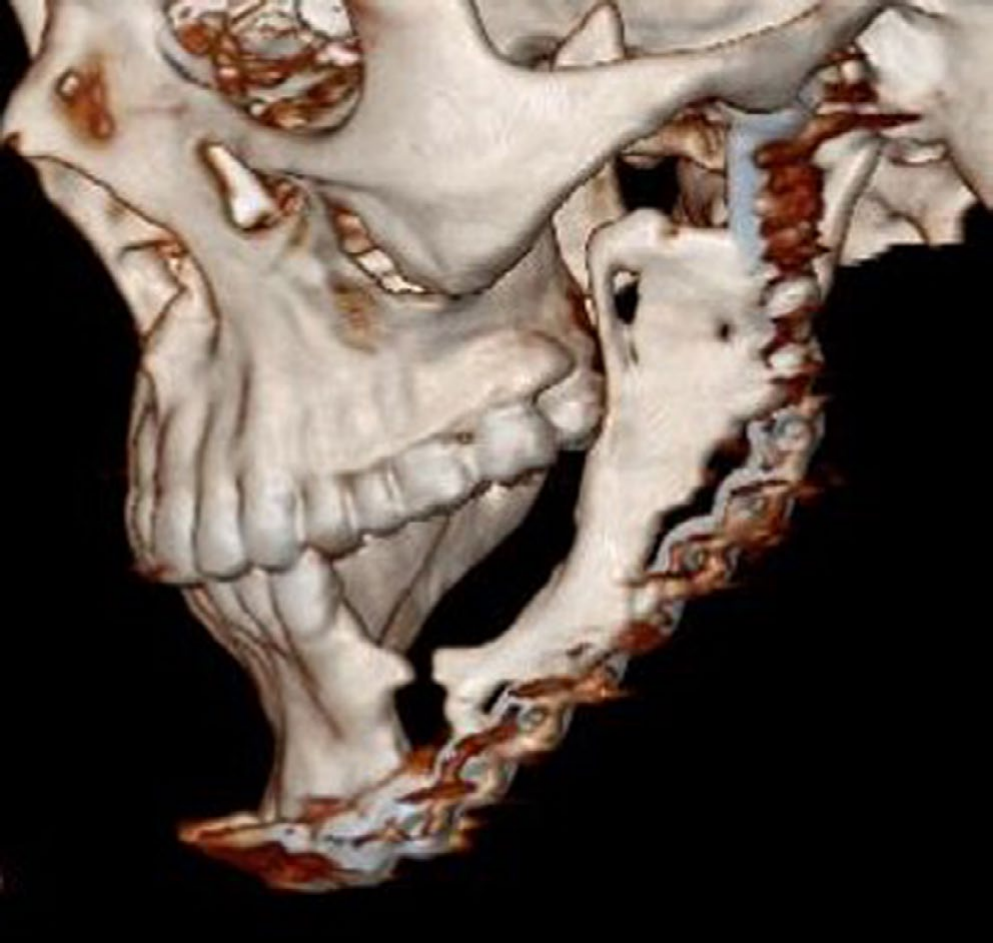
24‐mo postresection 3D CT scan view revealed new bone formation over resection area. The condyloid and coronoid‐like structures are detectable

**Figure 4:**
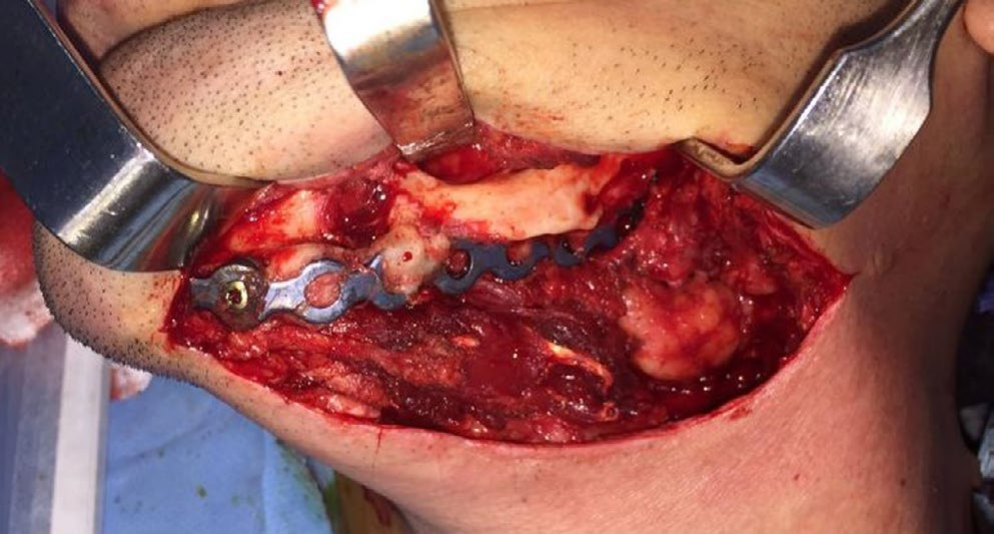
Intraoperative view of the new regenerated bone

Twenty‐four months later, mandibular reconstruction surgery with autogenous iliac bone graft was scheduled for subsequent dental implant rehabilitation. Radiographic examination at this stage demonstrated evidence of new bone formation over resection area. 3D CT scan views showed condyloid and coronoid‐like structures posteriorly and a strut of bone anteriorly (Figure 3). The same approach through previous scar was used to expose the new regenerated bone (Figure 4). The bone in mandibular body was resected and replaced with a corticocancellus bone block, harvested from left anterior iliac crest of the patient. The autogenous tricortical bone block was approximately 5 × 3 × 2 cm in size. The iliac graft was perforated and fixed to existing reconstruction plate using three biocritical screws. The resected regenerated bone was sent for histopathological study. Both submandibular and iliac incisions were closed in layers. Hemovac drain was inserted into the iliac wound and fixed to the skin. The patient made an uneventful postoperative recovery. Result of histopathological study confirmed the bony nature of the specimen.

## 5 DISCUSSION

Spontaneous bone regeneration (SBR) is a rapid, uncommon, and unexpected formation of new bone in a previous bone defect.^7^ The mechanism of spontaneous regeneration of the bone is not fully understood^8^; however, several factors have been suggested to influence this phenomenon. Most cases of this phenomenon reported in the literature are in younger individuals.^7^, ^8^, ^9^ Better bone regeneration in young patients is associated with higher cellular activity including bone absorption and regeneration. Higher cellular content and abundant mesenchymal cells differentiating into osteogenic cells are another potential theory in younger patients (Ref. [7] [p227], [9] [275]).

However, as described in this study, few numbers of older patients with spontaneous bone regeneration have been also reported in literature. This suggests that age may play an important role in spontaneous regeneration yet the possibility of this phenomenon is still potent throughout the lifetime and is not limited by age of the individual (Ref. [1] [p5]).

It is believed that the periosteum is a source of osteoprogenitor cells, and preservation of the periosteum is crucial for spontaneous regeneration potential.^9^ The presence of an intact periosteum or at least a part of it is an often‐documented factor for this phenomenon (Ref [7] [p227].

Low‐grade infection is believed to activate osteoblasts originating from the intact periosteum (Ref [8] [p155]). In present study, no sign of infection was encountered during the pre‐op and post‐op course of treatment.

Immobilization of the remaining segment has been suggested to promote osteogenesis.^10^ In present case, the remainder parts of the mandible were stabilized by a reconstruction plate.

## 6 CONCLUSION

This report describes a case of bone regeneration in a 21‐year‐old male who had mandibular resection for an extensive case of odontogenic myxoma.

It is important to notice that even in cases with bone regeneration, secondary bone grafting will still be required for future dental rehabilitation. Further studies in genetic and tissue engineering area are needed to make this spontaneous human body response, more efficient and predictable.

## CONFLICT OF INTEREST

None declared.

## AUTHOR CONTRIBUTIONS

Mahboube Hasheminasab: Have made substantial contributions to conception and performed the surgery. Reza Sharifi: Have made substantial contributions to conception and designed the article. Mahsa Mortazavi: Been involved in drafting the manuscript. Arezoo Javani: Been involved in drafting the manuscript and revising it critically for important intellectual content and given final approval of the version to be published.
